# Structural and functional analysis of the human POT1-TPP1 telomeric complex

**DOI:** 10.1038/ncomms14928

**Published:** 2017-04-10

**Authors:** Cory Rice, Prashanth Krishna Shastrula, Andrew V. Kossenkov, Robert Hills, Duncan M. Baird, Louise C. Showe, Tzanko Doukov, Susan Janicki, Emmanuel Skordalakes

**Affiliations:** 1 The Wistar Institute, 3601 Spruce St, Philadelphia, Pennsylvania 19104, USA; 2 Department of Biochemistry and Biophysics, Perelman School of Medicine, University of Pennsylvania, Philadelphia, Pennsylvania 19104, USA; 3 Division of Cancer and Genetics, School of Medicine, Cardiff University, Heath Park, Cardiff CF10 3AT, UK; 4 Stanford Synchrotron Radiation Lightsource, SLAC National Accelerator Laboratory, Stanford University, Menlo Park, California 94025, USA

## Abstract

POT1 and TPP1 are part of the shelterin complex and are essential for telomere length regulation and maintenance. Naturally occurring mutations of the telomeric POT1–TPP1 complex are implicated in familial glioma, melanoma and chronic lymphocytic leukaemia. Here we report the atomic structure of the interacting portion of the human telomeric POT1–TPP1 complex and suggest how several of these mutations contribute to malignant cancer. The POT1 C-terminus (POT1C) forms a bilobal structure consisting of an OB-fold and a holiday junction resolvase domain. TPP1 consists of several loops and helices involved in extensive interactions with POT1C. Biochemical data shows that several of the cancer-associated mutations, partially disrupt the POT1–TPP1 complex, which affects its ability to bind telomeric DNA efficiently. A defective POT1–TPP1 complex leads to longer and fragile telomeres, which in turn promotes genomic instability and cancer.

## Abstract (web summary)

POT1 and TTP1 are part of the shelterin complex that caps and stabilizes the ends of telomeres. Here the authors present a structural analysis of the human POT1-TTP1 complex, shedding light on how it assembles and how cancer-associated mutations impact its assembly and function.

Shelterin is a hexameric nucleoprotein complex responsible for maintaining the integrity of the ends of our chromosomes, known as telomeres^1^. Human shelterin consists of TRF1, TRF2, TIN2, RAP1, POT1 and TPP1 (refs 1, 2, 3), binds double and single-stranded telomeric DNA and is involved in a wide range of functions^4^,^5^. It suppresses DNA damage response by capping and protecting the ends of chromosomes from being recognized as DNA double-strand breaks^6^,^7^. It prevents exonuclease degradation by sequestering the telomeric overhang^8^. It also controls telomere length by regulating access of telomerase to the telomeric overhang^9^.

POT1 has a wide range of functions at telomeres all of which are geared toward maintaining the integrity of the telomeric overhang. POT1 binds single-stranded, telomeric DNA with high affinity and specificity^4^,^10^,^11^. POT1-DNA binding sequesters the telomeric overhang, thus assisting in telomere capping, downregulation of telomere elongation and ATR dependent DNA damage response^12^,^13^,^14^,^15^. POT1 telomeric DNA binding is mediated by the two N-terminal OB-folds of the protein, while the C-terminal portion of the protein binds TPP1 (refs 10, 16). Human and *S. pombe* TPP1 binding to POT1 enhances its DNA binding properties by 10-fold^9^,^11^,^17^,^18^. During the S-phase of the cell cycle, the human POT1–TPP1 complex recruits telomerase to telomeres^9^,^16^,^19^ via direct contacts of telomerase with the TEL patch located at the N-terminal OB-fold of TPP1 (ref. 20). Binding of POT1 to the telomeric overhang resolves G-quadruplexes and allows for telomerase loading to telomeres for telomere elongation^21^.

It was recently discovered that POT1 is frequently mutated in chronic lymphocytic leukaemia, familial melanoma and glioma^22^,^23^,^24^,^25^,^26^,^27^. There are currently 137 naturally occurring mutations of POT1 reported to be associated with human disease (cBioPortal for Cancer Genomics). Many of these mutations localize at the N-terminal OB-folds and disrupt POT1-DNA binding, while others are located at the C-terminus of the protein and were TPP1 binds^22^,^23^,^24^,^25^. POT1 N-terminal mutations primarily disrupt DNA-binding and are associated with chromosomal abnormalities such as irregular telomere length, fragile telomeres and chromosome end-to-end fusions, phenotypes usually associated with telomere uncapping^22^,^23^,^24^,^26^,^27^. However, the precise role of human POT1 C-terminal (POT1C) mutations in human disease is currently unclear.

Here, we investigate the mechanism of POT1–TPP1 assembly and how naturally occurring POT1 mutations contribute to cancer. Our data shows that POT1C consists of two domains, an OB-fold and a holiday junction resolvase (HJR) domain both of which make extensive interactions with TPP1 forming a tight heterodimer. Inspection of the structure reveals that several of these mutations either perturb the POT1C fold and/or disrupt POT1–TPP1 binding. Altering the natural state of the POT1–TPP1 complex affects the integrity of the telomeric overhang, leading to chromosomal abnormalities associated with a dysfunctional telomere capping complex leading to genomic instability and cancer.

## Results

### Structure of the human POT1–TPP1 complex

**Figure 1:**
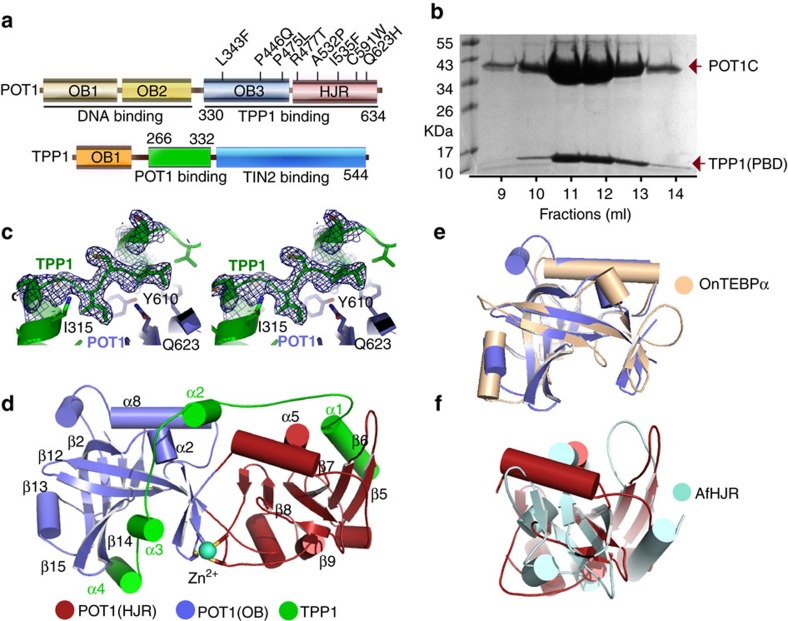
Crystal structure of the human telomeric POT1–TPP1 complex. (**a**) Primary structure of POT1 and TPP1 showing domains and their functional properties. POT1C disease mutations (L343F, P446Q, P475L, R477T, A532P, C591W and Q623H) discussed in this manuscript are also indicated. (**b**) SDS–page gel of the purified POT1C and TPP1(PBD) proteins used in structural and biochemical studies. (**c**) Stereo image of a portion (TPP1 bound to the OB-fold of POT1) of the simulated annealing omit map at 1 sigma contour level. (**d**) X-ray Crystal structure of the human POT1C—TPP1(PBD) complex; The OB-fold and HJR of POT1C are shown in blue and red colours respectively; TPP1(PBD) is shown in green colour. The Zn^2+^ ion coordinated by 4 cysteins (C382, C385, C503 and C506—stick) is shown as a cyan sphere. (**e**) Overlay of the POT1C and the *Oxytricha nova* telomere end binding protein alpha subunit (OnTEBPα—beige colour, PDB ID: 1OTC (ref. 28)) OB folds. (**f**) Overlay of the POT1C HJR (red colour) with its closest structural homologue *Archaeoglobus fulgidus* HJR (AfHJR—cyan colour; PDB ID: 2WIW).

We generated the interacting domains of human POT1 and TPP1 by limited proteolysis and mass spec analysis of the full length POT1–TPP1 complex (Fig. 1a,b). POT1C consists of residues 330–634 and TPP1 255–337 (TPP1(PBD)) (Fig. 1a,b). We solved the structure by the single wavelength anomalous dispersion (SAD) method and a Hg derivative (Table 1). The map showed clear electron density for POT1C residues 332–633 and TPP1(PBD) residues 266–326 (Fig. 1c).

POT1C consists of a classic OB-fold and a holiday junction resolvase domain (HJR) (Fig. 1d). The POT1C(OB) is a canonical OB-fold and is structurally most similar to the *Oxytricha Nova* Telomere End-Binding Protein (TEBPα, PDB ID: 1OTC—RMSD=2.2 Å—Fig. 1e). It is worth noting that an overlay of the TEBP alpha and beta dimer (PDB ID: 1OTC (ref. 28)) with that of the POT1–TPP1 structure shows no similarities in the organization of the two heterodimers. The organization of the six beta strands of the OB-fold forms a deep and well defined indentation on the surface of the protein, which comprises the canonical binding pocket of an OB-fold (Fig. 1d). Interestingly, the HJR is an insertion of the OB-fold and comprises residues P392-L538. The HJR is structurally similar to the *Archaeoglobus fulgidus* resolvase domain (AfHJR, PDB:ID 2WIW) with an RMSD 2.7 Å (Fig. 1f). HJR consists of seven antiparallel beta-strands surrounded by five alpha helices (α3, 4, 5, 6, 7) (Fig. 1d,f). Structural comparison of POT1C (HJR) with the AfHJR domain highlighted two distinct differences between these two HJR domains. One difference lies with the organization of the helices present in both HJR domains with four out of the five helices not overlapping (Fig. 1f). Another striking difference between the two HJR domains lies with the DNA binding pocket of the HJR. In AfHJR double stranded DNA binding is mediated primarily by two short antiparallel β-strands located on the surface of the protein and away from the five β-strands that form the core of the domain. In HJR, these two strands have shifted by a 50° rotation and 18 Å translation and are an integral component of the beta-sheet generated by all seven β-strands of this domain (Fig. 1f). The HJR putative DNA binding pocket is occupied by helix α3, the N-terminal helix of HJR. Extensive interactions between the two POT1C domains generates a long, almost cylindrical structure providing an extensive surface area for TPP1 binding. Further stabilization of the bilobal POT1C structure is mediated by a Zn^2+^ ion coordinated, tetra-cysteine cluster (C382, C385, C503, C506) locate at the interface of the two domains (Fig. 1d).

**Figure 2:**
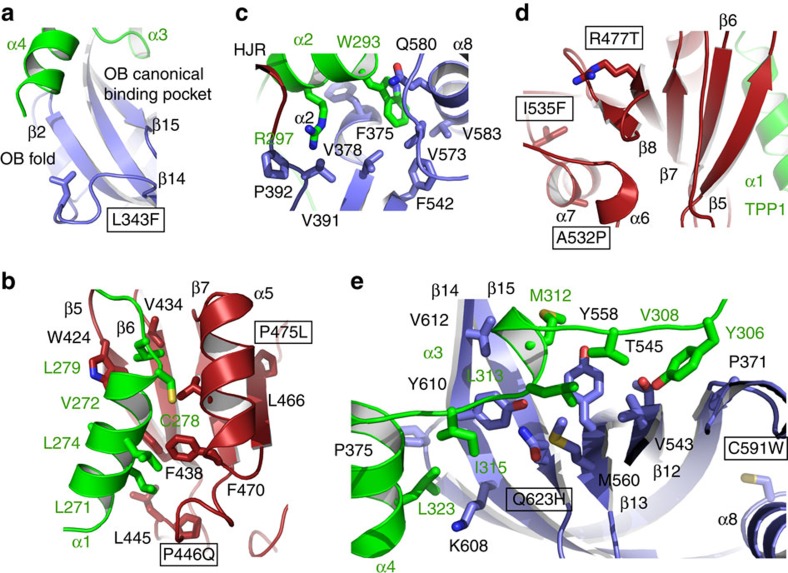
POT1C—TPP1(PBD) interactions. (**a**) Contacts between the POT1C OB-fold (blue) and the α3 and α4 helices of TPP1(PBD) (green); the disease mutation L343F is shown in stick. (**b**) Extensive interactions between the POT1C-HJR (red) and the leucine rich α1 of TPP1(PBD) (green) are shown. The P446Q and P475L mutations are highlighted with a box. (**c**) Contacts of the TPP1 helix α2 at the interface of the OB-fold and HJR of POT1C are shown. (**d**) The R477T, A532P and I532F disease mutations form part of the HJR of POT1C. The position of these amino acids with respect to the nearest TPP1 structural element (α1–green colour) are shown. (**e**) Extensive interactions between the POT1C OB-fold (blue) and helices α3 and α4 of TPP1(PBD) (green) are shown. The disease mutations C591W and Q623H are highlighted with a box.

TPP1(PBD) is an extended coil with four alpha helices distributed throughout the protein. The TPP1 polypeptide spans the entire length of POT1C and makes extensive contacts with both of its domains (Fig. 1d). Interestingly, TPP1(PBD) is organized in the opposite orientation (N—to—C-terminal) to that of POT1C so that the N-terminal portion of TPP1(PBD) (α1) interacts with the HJR while the C-terminal one interacts with the OB-fold. In particular, α1 of TPP1(PBD) makes extensive interactions with the surface of the β-sheet generated by the β-strands of the HJR. It also interacts with helix α5 of POT1C, which is located on the same face of the β-sheet of HJR and in parallel orientation with the β-strands forming the β-sheet (Fig. 1d). The TPP1(PBD) helix α1 is leucine/valine rich and the majority of contacts with the POT1C, HJR are hydrophobic in nature (Fig. 2a). TPP1, Leu271 is buried in the large hydrophobic pocket formed by L445, F438 and F470 of POT1C. The side chain of TPP1, L274 stacks against the side chain of POT1C, F470. TPP1, V272 and A275 are coordinated by the side chains of POT1C, F438 and W424. W424 of POT1C also makes a productive hydrogen bond with E278. L279 is buried in a well-defined hydrophobic pocket formed on the surface of POT1C by residues W424, V436, L466, V434 and the aliphatic portion of the side chain of E462. L281 is also buried within this pocket of POT1 but the contacts are limited to the side chains of W424 and T426.

Contacts between the loop that connects the TPP1(PBD) helices α1 and α2 and POT1C are limited to residues C285 and P288. Both of these residues make minor hydrophobic interactions with the OB-fold of POT1C. The loop is also held in place by a 2.7 Å hydrogen bond between the backbone of this loop and E394 of POT1C.

The TPP1(PBD) helix α2 localizes at the interface of the POT1C, OB-fold and HJR domains, where it is involved in extensive and specific interactions with the protein (Fig. 2b). Of note are the contacts generated by the highly conserved W293 and R297 of TPP1. The large W293 side chain is buried in a deep hydrophobic pocket formed by the POT1 residues F542, V583, V573, L574, F542, V378 and the aliphatic portion of Q580, which comprise part of helices α2, α7 and α8 of the OB-fold. The side chain of POT1, Q580 also hydrogen bonds the TPP1 α2 backbone and the side chain of H292. R257 of TPP1 also localizes at the interface of the OB and HJR of POT1C but unlike W293, its side chain is involved in a network of interactions with POT1C via solvent molecules (Fig. 2d). The only direct contact between R257 and POT1C involves a hydrogen bond with the backbone of V391.

Additional contacts between TPP1 and the POT1C OB-fold are extensive and involve helices α3 and α4 and the coils that connect them (Fig. 2c). Both of these helices are docked into the canonical, binding pocket of the OB-fold. More specifically, the TPP1 Y306 side chain stacks against P371 of POT1C. The branched side chains of TPP1, V308 and L313, are buried into a greasy hydrophobic patch located at the base of the OB-fold's binding pocket. TPP1, I315 of the linker that connects α3 to α4 coordinates POT1 Y610, F625, and the aliphatic portion of the K608 side chain. Contacts between the C-terminal helix (α4) of TPP1 and POT1C are limited to L325 and the side chains of P357 and K608 of POT1C.

### Several POT1C mutations reduce POT1–TPP1 binding

There are several POT1C mutations (L343F, P446Q, P475L, R477T, A532P, I535F, C591W and Q623H) that are associated with either familial glioma, melanoma or CLL (refs 22, 23, 24, 25, 26, 27) (Supplementary Table 1). The majority of these mutations (L343F, P446Q, P475L, R477T and C591W) are somatic and specific to CLL (ref. 22), while A532P and Q623H are germline and specific to familial glioma and melanoma^24^. To determine the role of the POT1C disease mutations in POT1–TPP1 binding and complex assembly, we performed Isothermal Titration Calorimetry (ITC) measurements. The experiments were carried out using purified, wild type (WT) TPP1(PBD) (residues 255–337) and WT or mutant POT1C (residues 330–634) (Supplementary Fig. 1), the same constructs we used for the crystallization of the POT1C–TPP1(PBD) complex. The proteins were overexpressed in *Escherichia coli* and purified to homogeneity using three successive steps of purification as described in the methods section of the manuscript. All TPP1(PBD) and POT1C, WT and mutant proteins, except for I535F, overexpressed stably and in sufficient quantities for the proposed studies (Supplementary Fig. 1).

**Figure 3:**
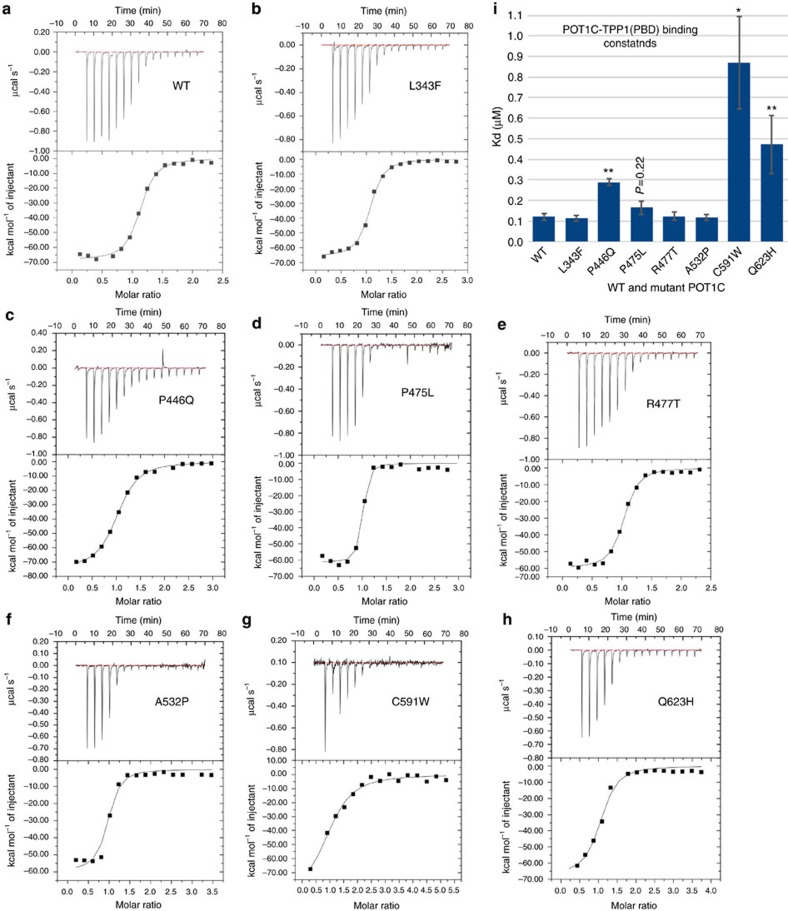
ITC binding data for the POT1C and TPP1(PBD) proteins. (**a**–**h**) ITC binding data of WT and mutant (L343F, P446Q, P475L, R477T, A532P, C591W and Q623H) POT1C with TPP1(PBD). (**i**) Bar graph of binding constants (Kd, μM) for WT and mutant POT1C proteins derived from the fitted ITC data. Three independent ITC experiments and a two-tailed Student's *t*-test was performed with respect to WT: *P<0.05, **P<0.01. The data clearly shows that the POT1C, P446Q, C591W and Q623H mutations reduce TPP1 binding.

Considering the extensive network of interactions between the two proteins it is not surprising that the binding constant of TPP1 for POT1 is in the nanomolar range (Kd=120±16 nM—Fig. 3b,f). Of the seven mutant POT1C proteins tested, only P446Q, C591W, and Q623H showed significant loss of TPP1 binding (Kds of 289, 870 and 471 nM, respectively—Fig. 3c,g,h,i; Supplementary Table 2). P475L displayed a marginal decrease in TPP1 binding (164±32 nM) but was not statistically significant (*P*=0.22). In contrast, L343F, R477T and A532P displayed almost WT TPP1 binding affinity (Kds 114, 123 and 117 nM, respectively—Fig. 3b,e,f,i and Supplementary Table 2). Consistent with the POT1C-TPP1(PBD) structure presented here, P446 and Q623 make direct contacts with TPP1, while the C591W mutation most likely displaces α8 of the POT1C(OB) (Fig. 2e), which indirectly affects TPP1 binding. P475 lies in the interior of the HJR and contributes to the fold of this domain. L343, A532 and R477 are located away from the TPP1 binding sites of POT1 and are either involved in the fold of the protein (L343, A532) or are solvent exposed (R477) and their function is currently unclear.

### POT1–TPP1 disruption does not alter telomerase processivity

**Figure 4:**
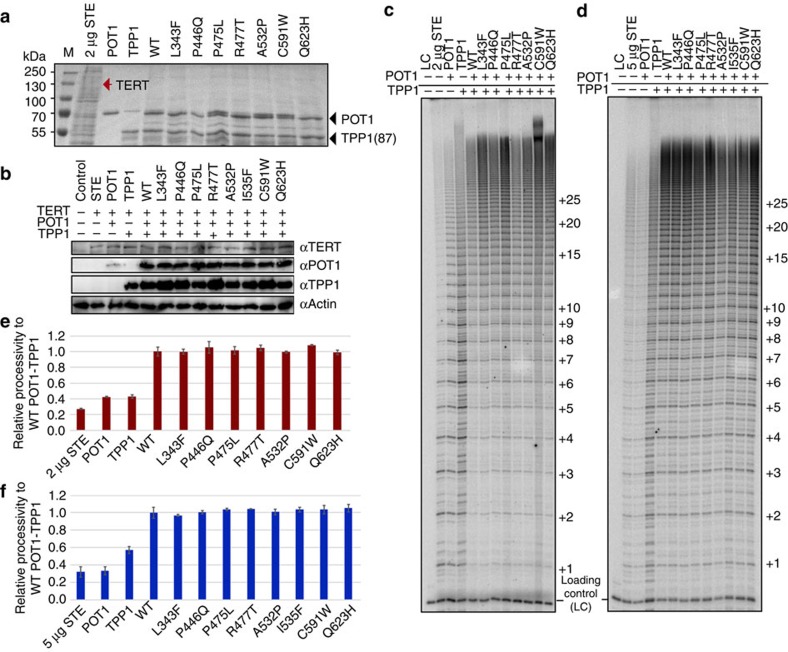
Telomerase direct activity assays. (**a**) SDS–PAGE gel of super-telomerase extract (STE) and *E. coli* purified full-length POT1 (flPOT1) and TPP1(87) proteins used in the direct assay of (**c**). The red arrow indicates where TERT is expected to run (∼130 kDa). The assay in **c** was carried out in the presence of 150 nM of purified POT1–TPP1 complex. (**b**) Western blot of HEK293T lysates used in the direct assay of panel (**d**). The gel shows the levels of transiently expressed POT1 and TPP1 are much higher than those of TERT, confirming that the direct assays were carried out at saturating levels of POT1 and TPP1. (**c**) Telomerase direct activity assay using 2 μg of STE; 150 nM purified WT or mutant (L343F, P466Q, P475L, R477T, A532P, I535F, C591W and Q623H) flPOT1; 150 nM WT TPP1(87); and 20 nM of primer A5 (TTAGGGTTAGCGTTAGGG). (**d**) Telomerase direct activity assay using 5 μg of STE co-transfected with WT or mutant (L343F, P466Q, P475L, R477T, A532P, C591W and Q623H) flPOT1 and WT full-length TPP1 (flTPP1). The STE was supplemented with 20 nM of A5 primer. The number of telomeric repeats added to the primer are indicated on the right of the gels (**c** and **d**,**e** and **f**) Quantification of telomerase processivity of panels (**c**) and (**d**) respectively. The values are the average of four independent experiments; error bars indicate s.d.

Since the POT1–TPP1 complex is directly linked to telomerase processivity^9^,^16^, we asked whether the POT1C mutations, associated with human disease, affect telomerase processivity. It is worth noting that the TEL patch of TPP1 implicated in telomerase binding is located at the N-terminus of TPP1. To address this question, we performed direct telomerase activity assays using cell extracts overexpressing super telomerase and at saturated levels of full length, WT or mutant POT1–TPP1. HEK293T cells extracts overexpressing human super-telomerase (the super-telomerase plasmids were a gift of the Lingner lab) were prepared as described by *Lingner et al*.^29^ We also overexpressed and co-purified the full length POT1 and an N-terminal truncation of TPP1 consisting of residues 87–544 ((TPP1(87)) (Fig. 1a). The two proteins were overexpressed separately in *E. coli* and the cells co-cracked to allow stable POT1–TPP1 complex formation prior to purification. We found that independent purification of the two proteins resulted in partial TPP1 degradation suggesting that TPP1 is not stable alone (Fig. 4a). We were unable to purify sufficient amounts of the full-length I535F POT1 mutant for this assay. In addition, we prepared HEK293T lysates overexpressing human super-telomerase, full-length POT1 and TPP1 as described by *Nandakumar et al*.^20^. We examined the stability and levels of WT and mutant POT1 and TPP1 proteins overexpressed in transfected HEK293T cells using western blot analysis. Western blot analysis shows that all proteins (WT and mutant POT1 and TPP1) express at levels similar to the WT protein (Fig. 4b).

Both approaches show robust telomerase activity with increased telomerase processivity in the presence of the WT POT1–TPP1 complex (Fig. 4c–f and Supplementary Fig. 2), consistent with what has been previously reported^9^. The POT1 mutants L343F, P475L, R477T, A532P and I535F showed WT telomerase processivity (Fig. 4c–f), in agreement with the ITC data, which shows that these mutant proteins bind TPP1 with WT binding affinity (Fig. 3). Unexpectedly, the P446Q, C591W and Q623H POT1 mutants, which bind TPP1 with 2–7 fold less affinity (Fig. 3c,g,h and i), also show WT telomerase processivity within the margin of error (Fig. 4c–f).

### Partial POT1–TPP1 disruption reduces POT1-DNA binding

**Figure 5:**
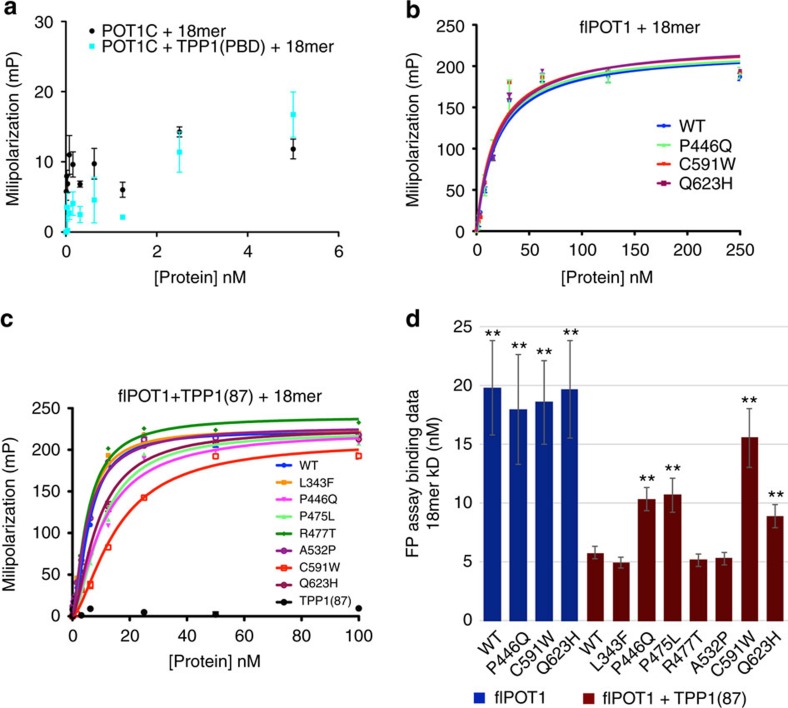
POT1–TPP1 telomeric DNA binding assays. (**a**) FP assays of POT1C and the POT1C–TPP1(PBD) complex with a single-stranded DNA probe consisting of 3 telomeric repeats (18mer). (**b**) FP assays of the WT and mutant (those that partially disrupt the POT1–TPP1 complex P446Q, C591W and Q623H) flPOT1 with the 18mer DNA probe. (**c**) FP assays of the WT and mutant flPOT1−TPP1(87) complex with the 18mer. (**d**) Bar graph showing the differences in Kd (nM) between the WT and mutant flPOT1 and flPOT1–TPP1(87) complex. The values are the average of three independent measurements and a two-tailed Student's *t*-test was performed with respect to WT POT1–TPP1(PBD) complex: *P<0.05, **P<0.01.

POT1 binds the telomeric overhang with high affinity and selectivity, a process enhanced by TPP1 binding^11^,^17^. For this reason, we asked if the POT1 disease mutants located in the C-terminal portion of the protein and where TPP1 binds, affect POT1-DNA binding. Fluorescence Polarization (FP) assays using a fluorescently labelled DNA probe consisting of three telomeric repeats (TTAGGG)_3_ (18mer) were carried out in the presence of (a) the POT1C and TPP1(PBD) protein complex used for crystal and ITC studies (Figs 1b and 3a) (b) full-length POT1 WT and three mutant (P446Q, C591W, and Q623H) proteins and (c) the *E. coli* purified, full-length WT and mutant (L343F, P446Q, P475L, R477T, A532P, C591W and Q623H) POT1 and the N-terminally truncated TPP1(87) used in the telomerase direct assays (Fig. 5a).

FP data show that POT1C alone and the POT1C-TPP1(PBD) complex do not bind single-stranded telomeric DNA (Fig. 5a). The full-length WT and mutant (P446Q, C591W and Q623H) POT1 proteins alone all bind single-stranded telomeric DNA with a Kd of ∼20 nM (Fig. 5b,d; Supplementary Table 3). For the POT1–TPP1 complex our data shows that the WT complex binds the telomeric overhang with approximately 5.8±0.5 nM (Fig. 5c,d and Supplementary Table 3), consistent with what's been reported previously^4^,^9^. Interestingly, the P446Q, P475L, C591W and Q623H mutants show a decrease in DNA binding affinity (Kd=10.3±1.0 nM, 10.7±1.4 nM, 15.6±2.5 and 8.9±1.0 nM respectively—Fig. 5c,d and Supplementary Table 3) in agreement with the ITC data, which shows that these POT1 mutant proteins partially disrupt the POT1–TPP1 complex (Fig. 3 and Supplementary Table 2). In addition, the L343F, R477T and A532P POT1 mutants, which have wild type TPP1 binding affinity (Fig. 3 and Supplementary Table 2) also bind telomeric DNA with wild type affinity (Fig. 5c,d and Supplementary Table 3).

### POT1C mutant proteins localize to telomeres

**Figure 6:**
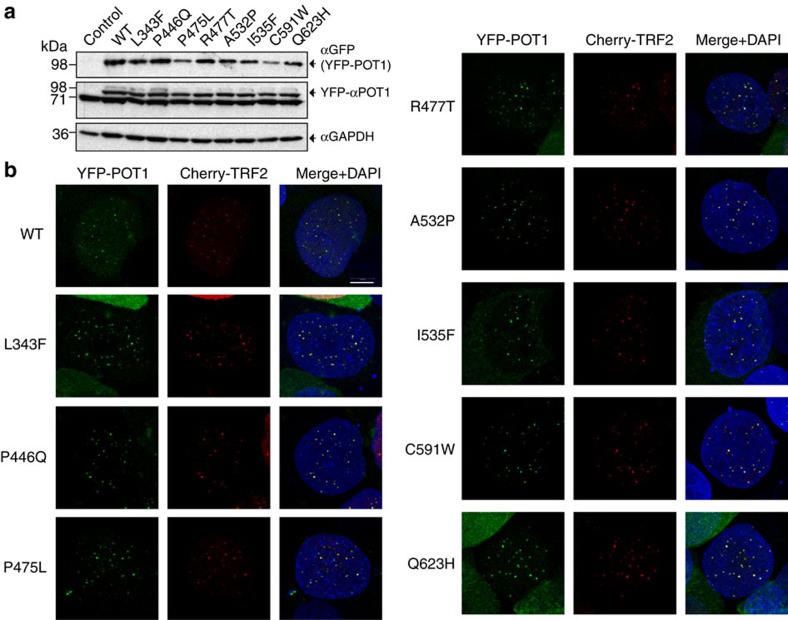
Cell imaging for localization of WT and mutant POT1 to telomeres. (**a**) Western blot showing the levels of YFP tagged, WT and mutant (L343F, P466Q, P475L, R477T, A532P, I535F, C591W and Q623H) full length POT1. We used GFP (αGFP) or POT1 (αPOT1) to detect the levels of YFP-POT1 in each cell line. The band at 71 kDa and below the YFP-aPOT1 is a non specific protein. GAPDH was used as a loading control. (**b**) Maximum projection images of co-localization of YFP-POT1 (green) and Cherry-TRF2 (red) proteins are shown. Merged images include DAPI. Scale bar, 5 μm. The data clearly shows co-localization of WT and mutant flPOT1 proteins to telomeres.

To determine the impact of the POT1C mutations on telomere targeting, we co-expressed YFP-tagged POT1 with mCherry-tagged TRF2 in HEK293T cells and used confocal imaging to examine their localization. HEK293T cells were transiently transfected with Cherry-TRF2 and YFP-POT1 and fixed 24 h later. Western blot analysis of whole-cell lysates of transfected HEK293T cells showed overexpression of the WT and mutant YFP-POT1 proteins (Fig. 6a). All of the POT1mutants co-localized with Cherry-TRF2, similar to wild type, which indicates that the mutations do not prevent POT1 telomere targeting (Fig. 6b).

### Partial POT1–TPP1 disruption leads to longer telomeres

**Figure 7:**
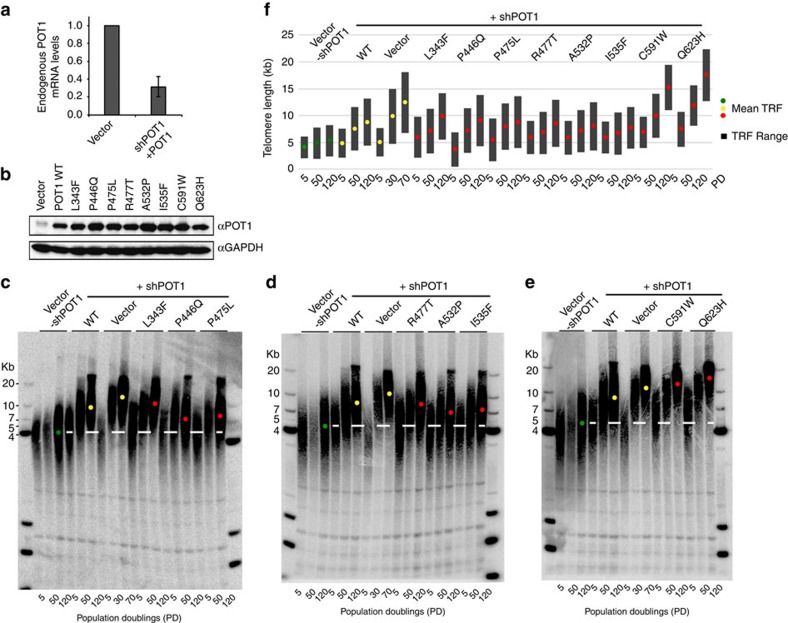
Southern blot analysis of WT and mutant POT1. (**a**) Quantitative RT-PCR showing knock down of endogenous POT1 mRNA, normalized to GAPDH transcript levels, after lentiviral infection of HEK293T cells with shPOT1 and WT Flag-POT1; selection was carried out with puromycin and blasticidin;±s.d. (*n*=3). (**b**) Western blot showing WT and mutant POT1 protein expression levels in HEK293T cells expressing shPOT1 and Flag-POT1. Cells infected with the empty pLU and pLKO.1 lentiviral vectors are used as controls. (**c**,**d**,**e**) Southern blot of HEK293T cells expressing shPOT1 and WT or mutant flPOT1. DNA from 5, 50 and 120 populations doublings (PD) is shown. DNA length standards are indicated along the left and right of the gels. The white dashed line indicates the average telomere length of HEK293T cells transfected with the vector alone and no shPOT1 after 5, 50 and 120 PD. The vector (- shPOT1), vector (+shPOT1) and WT POT1 (+shPOT1) controls are indicated with a green and yellow dot respectively. The POT1 mutants (L343F, P466Q, P475L, R477T, A532P, I535F, C591W and Q623H) are indicated with red dots. (**f**) Quantification of the mean telomere length (kb) from panels (**c**,**d** and **e**). The same colour scheme as in panels **c**,**d** and **e** is used. Black bars indicate the range of telomere restriction fragments (TRF).

To determine if the POT1 cancer mutations maintain telomere length homoeostasis, we carried out southern blot analysis of WT and mutant POT1 transfected HEK293T cells. Stable HEK293T cell lines expressing Flag-POT1 were prepared using lentiviral infection. We reduced the levels of endogenous POT1 using a shRNA (shPOT1) targeting its 3′UTR region (Fig. 7a). We also generated two mock-treated control cell lines by co-infecting HEK293T cells with the vector (not carrying the POT1 gene) and with or without the shPOT1.

We confirmed similar levels of exogenous WT and mutant POT1 protein expression in the presence of shPOT1 in all cell lines using western blot analysis (Fig. 7b). We find that the length of telomeric DNA for the vector alone (without shPOT1) remains almost the same even after 70 population doublings (PD). In contrast, the vector alone and the WT POT1 cell lines treated with shPOT1 have significant longer telomeres after 50 and 120 PD. More specifically, the average telomere length for the vector alone without shPOT1 is ∼4.5 Kb. The telomere length of cells transfected with the vector or WT POT1 and shPOT1 after 120 PD is ∼12.5 and 8.5 Kb, respectively. The cell lines transfected with the POT1 mutants that partially disrupt the POT1–TPP1 complex and shPOT1 also have longer telomeres after 50 and 120 population doublings compared to the vector alone without shPOT1 (Fig. 7c–f). The POT1 mutants P446Q, C591W and Q623H, which partially disrupt the POT1–TPP1 complex showed the most significant change in telomere length. Approximately 4.5, 10 and 12.5 Kb increase in telomere length is observed for P446Q, C591W and Q623H respectively (Fig. 7c–f), while L343F, P475L, R477T, A532P and I535F show 3–5 Kb increase in telomere length (Fig. 7c–f).

### Partial POT1–TPP1 disruption leads to fragile telomeres

To better understand the role of the POT1 cancer-associated mutations in telomere maintenance, we examined the phenotype of HEK293T cells infected with WT or mutant POT1 (same as those used for Southern blot analysis, Fig. 7b) using fluorescence *in situ* hybridization (FISH). The endogenous POT1 in these cell lines was reduced by infection with shPOT1 (Fig. 7a). We prepared metaphase spreads of these cell lines by fixing the chromosomes to microscope slides with formaldehyde, and hybridizing telomeres with a 5′ TelC-Tamra peptide nucleic acid (PNA) probe. Chromosomal DNA was stained with DAPI prior to imaging.

**Figure 8:**
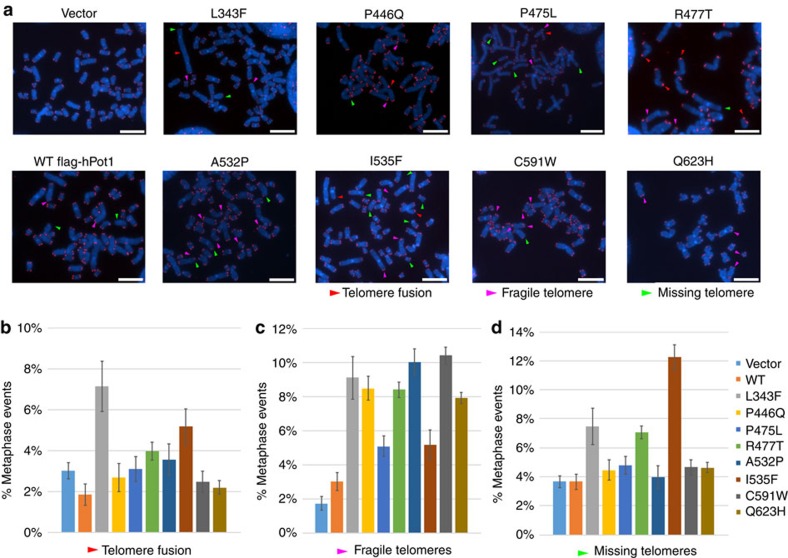
Fluorescence *in situ* hybridization data. (**a**) Telomeric FISH metaphase spreads of cells expressing WT and mutant (L343F, P466Q, P475L, R477T, A532P, I535F, C591W and Q623H) flPOT1 proteins after 50 population doublings (Red, TelC-Tamra; blue, DAPI). Endogenous POT1 levels were reduced with shPOT1. Telomere fusions, fragile telomeres and missing telomeres are indicated by red, pink and green arrows respectively. Scale bar is 5 μm. (**b**-**d**), Quantification of telomere fusions, fragile, and missing telomeres from the metaphase spreads of (**a**). Bars indicate the per cent of metaphase events. Error bars are indicating s.d. An average of 500 chromosomes were counted in each experiment.

We counted the frequency of chromosome fusions, fragile telomeres, and telomere free ends 50 population doublings after infection. HEK293T cells carrying the empty vector showed an average of 2–3% of chromosomes with chromosome fusions, fragile and missing telomeres, similar to that of the cell line overexpressing WT POT1. Elevated levels of fusions were observed for the L343F (7%) and I535F (5%) POT1 mutants, respectively (Fig. 8a,b). Interestingly, all POT1 mutants showed a significant increase of fragile telomeres (L343F 9%; P446Q, R477T and Q623H, 9%; P475L and C591W 10%) compared to ∼3% for the empty vector and WT POT1 (Fig. 8a,c). We also observed elevated levels of missing telomeres in cells overexpressing L343F, R477T (7%) and I535F (12%) POT1 mutants (Fig. 8a,d).

## Discussion

The cancer phenotypes associated with POT1C naturally occurring mutations are diverse (familial melanoma, glioma and CLL (refs 22, 23, 24, 25, 27), Supplementary Table 1), which points to the complex nature and function of the telomeric complex POT1–TPP1 at telomeres. N-terminal, POT1 disease mutations disrupt POT1-DNA binding and release the telomeric overhang, which results in persistent telomere replication by telomerase^22^,^23^,^24^,^25^. There is no evidence currently that supports POT1C or POT1C-TPP1(PBD), DNA binding (Fig. 5a). However, POT1C interacts with TPP1, and POT1 mutations that disrupt the complex would be expected to influence its functions, which include localization to telomeres, stimulating telomerase processivity, and enhanced POT1 DNA binding activity^4^,^5^,^9^. Moreover, the fact that a POT1 mutation confers a telomere instability phenotype, over a long period of time might provide the genetic variation that is required for clonal evolution. This would be even more apparent in tumours with a high-tumour burden, such as CLL, where the total number of cells will be in the 10–100 s of billions range. Thus, a combination of the long time it takes for these tumours to evolve and the tumour burden means that even subtle defects in telomere stability could have a dramatic effect on long-term clinical outcome.

Our data on several of the referenced disease associated POT1 mutations supports the above hypothesis. Cell imaging shows that all reported POT1 mutant proteins localize to telomeres (Fig. 6). Despite the fact that all POT1 mutant proteins localize to the telomeric overhang, FP assays show that only the mutants (P446Q, C591W and Q623H) that partially disrupt the POT1–TPP1 complex display significantly lower affinity for telomeric DNA (Figs 3 and 5c,d). Reduced affinity of the POT1–TPP1 complex for the telomeric overhang will result in persistent telomere elongation by telomerase^30^. Persistent telomerase action at the end of chromosomes will generate longer telomeres than those observed for the WT POT1–TPP1 complex (Fig. 7c,d). This observation is consistent with the Southern blot analysis presented here, which shows that HEK293T cells transfected with the POT1C mutants have longer telomeres than the cells transfected with WT POT1. This observation is particularly distinct for the POT1 mutants that partially disrupt the POT1–TPP1 complex with ∼4.5, 10 and 12.5 Kb increase in telomere length for P446Q, C591W and Q623H respectively (Fig. 7). It has also been well established that unregulated telomere length results in chromosomal abnormalities associated with telomere signal free ends and fragile telomeres^31^,^32^,^33^. Consistent with this hypothesis POT1 mutations that disrupt the POT1–TPP1 complex show elevated levels of missing and fragile telomeres (Fig. 8). This defect is particularly prominent when it comes to fragile telomeres with an average of 10% of fragile telomeres for all POT1 mutants when compared to ∼3% for the empty vector and WT POT1. Interestingly, none of the disease mutations appear to affect telomerase processivity (Fig. 4c–f).

We show that several of the referenced POT1 mutations (P446Q, C591W and Q623H) partially disrupt the POT1–TPP1 complex (Fig. 3c,d,g–i). This observation is in agreement with the POT1–TPP1 structure (Fig. 2), as well as data previously reported by *Liu et al*.^3^, which shows that the 417–445 and 616–628 of POT1 peptides make direct contacts with TPP1. P446Q is critical for TPP1 binding as it comprises part of the loop that connects the HJR strands β6 and β7 and coordinates L271 of the N-terminal helix α1 of TPP1 (Fig. 2b). The P475L mutant exhibits marginal disruption of the POT1–TPP1 complex forms part of β8 located at the core of HJR domain and contributes to the fold of this domain (Fig. 2b). The proline to leucine change most likely subtly perturbs the organization of the structural elements of the HJR domain surrounding this residue including the re-organization of helix α5, which makes extensive interactions with α1 of TPP1, thus affecting POT1–TPP1 binding (Fig. 2b). C591W is located at the C-terminus of helix α8, which spans the entire length of the side of the OB-fold and plays a critical role in the organization of this domain of POT1C (Fig. 2e). Moreover, the N-terminal portion of helix a8 makes extensive interactions with helix α2 of TPP1 and in particular the side chain of W293. Displacement of the POT1 helix α8 would lead to re-organization of this region of POT1C(OB), thus affecting TPP1 binding. The POT1–TPP1 structure also shows that Q623 directly engages TPP1 (Fig. 2e). The Q623 residue is located at the heart of the canonical binding pocket of the POT1C OB-fold, and the solvent accessible side chain coordinates the backbone of helix α3 of TPP1. More specifically, the NE2 of Q623 makes a productive hydrogen bond with the carboxyl of L313 of TPP1. Introduction of the larger hydrophobic side chain in this position would lead to loss of this hydrogen bond and rearrangement of the helix from its current position. Structural reorganization of helix α3 would affect the extensive network of interactions mediated between the residues V308, L313 and I315 of helix α3 and its connecting loops of TPP1 with V543, Y558, M560, Y610 and F625 of the POT1C OB-fold, thus affecting POT1–TPP1 binding.

Unlike P446Q, C591W and Q623H, the L343F, P475L, R477T and A532P POT1C mutants displayed WT, TPP1 and DNA binding affinity (Figs 3 and 5). L343F comprises part of the loop that connects the POT1C, OB-fold, β-strand β1 to the α-helix α1. L343 is important for the structural organization of this loop and does not make direct contacts with TPP1 (Fig. 2a). Similarly, R477T is part of the β8 of HJR and is located at the opposite face of this domain and where helix α1 of TPP1 binds (Fig. 2d). Unlike L343, R477 is solvent exposed and does not make any contacts with TPP1; in fact, the nearest TPP1 contact point is ∼18 Å away. A532 is part of α7 of POT1(HJR) and like R477, is located on the opposite side of the HJR domain where helix α1 of TPP1 binds. I535 is located in α7 of the HJR domain with the hydrophobic side chain buried into the core of this domain. Introducing the larger phenylaline side chain would disrupt the core of the HJR domain and therefore lead to the destabilization of the protein (Fig. 2d). Interestingly, isolation of the A532P and in particular of I535F POT1C and flPOT1 mutant proteins resulted in lower levels of proteins compared to WT protein (Supplementary Fig. 1b,c). This finding suggests that the A to P and I to F amino acid changes of A532 and I535 residues most likely affects the stability of these mutant POT1 proteins. Isolation of the I535F protein did not produce adequate amounts of this polypeptide for TPP1 and DNA binding assays. However, these mutant proteins appear to be at WT levels in cell based assays allowing us to study their effect in telomerase activity, processivity, telomere localization and telomere defects including telomere length, and chromosomal abnormalities (Figs 4, 6, 7, 8). Southern blot analysis showed telomere length changes of ∼3–5 Kb—Fig. 7d,e. There is also a significant increase in telomere fusions, fragile and missing telomeres in the telomeric FISH staining for the I535F mutant (Fig. 8).

Taken together our data show that it is a confluence of factors that contribute to malignant effects associated with POT1C mutants. Some of these mutations (P446Q, C591W and Q623H) partially disrupt the POT1–TPP1 complex and the DNA binding affinity of POT1, which in turn affects the ability of the complex to regulate telomerase access to telomeres efficiently. Deregulation of telomerase activity at the chromosomal terminus would result in constitutive telomeric elongation and increased proliferative potential^23^. In part, POT1 regulates telomere length^34^,^35^,^36^,^37^, by regulating access of telomerase to telomeres, an effect modulated by TPP1 binding^9^,^21^,^38^,^39^. Partial disruption of the POT1–TPP1 complex could therefore lead to persistent access of telomerase to the telomeric overhang and result in longer, fragile telomeres.

Surprisingly, a set of the reported disease mutations (L343F, A532P and R477T) do not appear to have a significant effect in the assembly of the POT1–TPP1 complex as indicated by our structural and biochemical data (Figs 2, 3, 4, 5, 6, 7). It is however worth noting that all of these mutations show a significant increase in fragile telomeres with defects in telomere length, missing telomeres and chromosome fusions. The precise role of these mutations in POT1–TPP1 function are currently unclear and further studies are required to better understand their role in cancer.

Germline variants in POT1 have been detected in familial melanoma and glioma^22^,^23^,^24^,^25^. Somatic mutations have been identified in POT1 and has been identified as a susceptibility locus for CLL (ref. 40). Somatic mutations in POT1 have also been identified in CLL B-cell clones and whilst the impact of these mutations on telomere length has not been established they are associated with increased chromosomal instability^22^. It is worth noting that POT1 mutations drive carcinogenesis in melanoma, glioma and CLL patients usually in combination with an array of other defective genes, such as CDKN2A, CDK4, BAP1, Notch1, SF3B1, TP53 and ATM, which have already been identified to predispose patients to these malignant diseases. Stratification of CLL patients based on the telomere length of their CLL B-cell clones, reveals that those with short telomeres, within the length ranges in which telomere fusion can be detected, exhibit an extremely poor prognosis^41^. This is presumed to arise as a consequence of telomere driven genome instability, clonal evolution and tumour progression^27^. While the majority of patients with long telomeres exhibit a better prognosis and a more stable genome, a subset of patients still progress with their disease. It will thus be of interest to examine the relationship between somatic POT1 mutation, telomere length and chromosomal instability and how this impacts on disease progression.

## Methods

### Protein expression and purification

Human POT1C, comprising residues 330–634, was identified via limited proteolysis and cloned into a pET28b vector containing a N-terminal hexahistidine—pMocr fusion tag, cleavable by TEV protease. The TPP1(PBD) construct was designed to contain residues 255–337 and was cloned into a pET28b vector containing a N-terminal hexahistidine tag cleavable by TEV protease. Both POT1C and TPP1(PBD) were overexpressed in *E. coli* ScarabXpress T7 *lac* competent cells (Scarab Genomics) at 18 and 30 °C for 4 h, respectively, using 1 mM IPTG (isopropyl-β-D-thiogalactopyranoside; Gold Biotechnology). The cells were harvested by centrifugation and lysed in a buffer containing 25 mM Tris–HCl, pH 7.5, 1.0 M KCl, 1.0 M Urea, 5% glycerol, 1 mM phenylmethylsulfonyl fluoride (PMSF), and 1 mM benzamidine (Ni Buffer A) via sonication. The proteins were purified over a Ni-nitrilotriacetic acid (Ni-NTA - MCLab) column, buffer exchanged while on the Ni-NTA column with 25 mM Tris–HCl, pH 7.5, 0.2 M KCl and 5% glycerol (Ni Buffer C). The complex was eluted with 300 mM imidazole onto a tandem HS(poros)—HQ(poros) columns (Applied Biosystems) equilibrated with Ni Buffer C. The HS–HQ columns were then detached and the POT1C-TPP1(PBD) complex was eluted from the HQ column with a salt gradient of 0.2 M KCl to 1.0 M KCl. The fusion tags were cleaved by TEV overnight at 4 °C and removed from the sample by an additional step of Poros-HS and HQ. The clean complex was passed over a Superdex S200 (GE Healthcare) to remove any aggregates.

Full-length WT and mutant POT1 were cloned into the same vector as POT1C. TPP1(87–544) was cloned into a pET28b vector containing an N-terminal hexahistidine-MBP (maltose binding protein) cleavable by TEV protease. POT1 and TPP1 were overexpressed separately in ScarabXpress cells at 18 °C overnight using 1 mM IPTG. The cells were harvested by centrifugation and lysed in Ni buffer A via sonication. The proteins were first purified over a Ni-NTA column and then eluted onto an amylose column (New England Biotech) to further purify the POT1–TPP1 complex and remove excess POT1 before eluting with a buffer containing 25 mM Tris–HCl, pH 7.5, 0.5 M KCl, 5% glycerol, 1 mM DTT and 30 mM maltose. The fusion tags were cleaved with TEV protease overnight at 4 °C. The POT1–TPP1 complex was then buffer exchanged to remove the maltose and passed over an orthogonal Ni-NTA and amylose column to remove any residue fusion tags and TEV protease from the samples. The purified full-length POT1–TPP1 complex was then concentrated and ran on an SDS–PAGE gel with known concentrations of BSA standard and quantified using ImageQuant TL (GE Healthcare) to determine the concentration of the complex.

### Protein crystallization and structure determination

POT1C-TPP1(PBD), crystal screening produced a crystal hit under sitting-drop vapour diffusion at room temperature in a crystallization buffer containing 2.4 M KCl, 50 mM K/Na Tartrate, 20 mM BaCl_2_, and 0.1 M Sodium Citrate, pH 5.5. (A longer construct of POT1C consisting of residues 325–634 produced a different crystal form that belonged to the P1 space group and diffracted to 3 Å at best.) The new crystal form belongs to the P4_1_22 (sg91, 1 copy in asymmetric unit) and diffracted to 2.1 Å. The native data set (containing Zn) was collected from 2 crystals at 1.03317 Å wavelength at BL12-2 SSRL to 2.1 Å resolution. The radiation damage was slowed with a careful absorbed dose estimate, allowing high-multiplicity and accumulating a significant anomalous signal from the present Zn and Sulfurs in the protein. Both the native and derivative data were processed with XDS (autoxds script at SSRL) with a zero dose correction.

The structure was solved using a single methyl mercury (meHg) derivative using the SAD approach as implemented in SHELXCDE using the graphical interface of the HKL2MAP software. SHELXD identified five well defined Hg sites and SHELXE extended and optimized the initial phases to 2.1 Å and generated a preliminary structure of 330 residues with excellent contrast (0.943) and connectivity (0.843) resulting in FOM of 0.605. Subsequently the model was traced using 10 cycles of BUCCANEER. The resulting model was almost complete—358 sequenced residues. The remaining model was improved in COOT and refined with BUSTER (version 2.10.2). The refinement converged to *R*/*R*_free_=17.3/19.3% to 2.1 Å resolution. The model has excellent stereochemistry as examined by MOLPROBITY server (http://molprobity.biochem.duke.edu/).

### Isothermal titration calorimetry (ITC)

We carried out ITC experiments on a MicroCal iTC200 (Malvern) using separately purified TPP1(PDB) and WT and mutant POT1C proteins. The purified proteins were buffer exchanged into a buffer containing 25 mM Hepes, pH 7.5, 0.1 M KCl, 5% glycerol, and 1 mM TCEP. Protein concentration was measured using a Bradford Assay^42^. TPP1(PBD) at a concentration of 100 μM was injected into a cell containing 10 μM POT1C until saturation was reached. For the ITC experiment, the cell of the calorimeter was kept at 25 °C, and the volume of each injection was 2.47 μl with a total of 16 injections. Analysis of the ITC data used the Origin analysis software (GE Healthcare) to obtain binding constants and ratios.

### Fluorescence polarization (FP) assays

We performed FP DNA binding assays using an Envision Xcite Multilabel Plate Reader (Perkin Elmer). 20 μl binding reactions were carried out in a buffer containing 20 mM Hepes, pH 7.5, 100 mM KCl, 2 mM MgCl2, 1 mM EDTA, 2 mM DTT, 1 mg ml^−1^ BSA, 5% v/v glycerol and 75 nM polyT_50_ competitor (IDT). The 18mer DNA probe (TTAGGGTTAGGGTTAGGG) was purchased with a 5′ 6-FAM label from IDT. The final probe concentration used was 2.5 nM, while the POT1–TPP1 protein concentration ranged from 0 to 100 nM or POT1C-TPP1(PBD) concentrations ranged from 0 to 5 μM. The reactions were incubated at room temperature for 30 min and pipetted in triplicate into a black 384 well optiplate (PerkinElmer). The reactions were excited with 480 nm light and the emissions were measured at 535 nm light. The milipolarization (mP) values were calculated by the Envision operating software (PerkinElmer). The data was fit and the binding constants were determined with a one-site binding, nonlinear regression model using PRISM 5.0 (GraphPad Software, San Diego California USA, www.graphpad.com).

### Cell culture

The WT and mutant full-length human POT1 genes were cloned in the pLU-EF1A-iBlast (pLU) vector with an N-terminal 1xFlag tag and 4 μg of vector was used to transfect HEK 293T cells using lipofectomine 2000 (Invitrogen) to test expression. HEK293T cells were cultured in growth medium containing Dulbecco's modified Eagle medium (Cellgro) supplemented with 10% fetal bovine serum (Sigma) and 100 units ml^−1^ penicillin and 100 μg ml^−1^ streptomycin (Sigma). All human cells were cultured at 37 °C with 5% CO_2_ and harvested 48 h after transfection.

Stable HEK293T cell lines overexpressing 1xFlag-POT1 were carried out using lentiviral infection to deliver WT and mutant human POT1 genes in the pLU vector with blasticidin resistance. A pLKO.1 vector with puromycin resistance carrying the shRNA (TRCN0000009837) targeting the 3′UTR of endogenous POT1 (shPOT1) was obtained from the Sigma Mission shRNA library and was delivered with lentiviral infection. Lentiviral particles were prepared by lipofectomine 2000 (Invitrogen) transfection of HEK293T cells with the pLU or pLKO.1 and lentiviral production vectors. Growth media was spiked with 5 μg ml^−1^ blasticidin S, and 2 μg ml^−1^ puromycin.

### Western blot

For the western blot analysis of HEK293T cells co-transfected with hTERT, hTER, POT1 and TPP1, standard immunoblot protocols were used with the following antibody dilutions: anti-human TERT (abx120550, Abbexa, 1:1,000, dilution), anti-human POT1 antibody (ab21283, Abcam, 1:1,000 dilution), anti-human ACD (SAB2100024, Sigma, 1:1,000 dilution), anti-Actin antibody conjugated to HRP (A3854, Sigma, 1:1,000 dilution), and secondary HRP-conjugated anti-sheep IgG (sc-2924, Santa Cruz, 1:1,000) and anti-rabbit IgG (A0545, Sigma, 1:1,000 dilution) were used to detect the human TERT and human POT1 and TPP1 antibody, respectively.

For western blot analysis of HEK293T cells stably expressing shPOT1 and WT and mutant POT1 proteins, standard immunoblot protocols were used with the following antibody dilutions: anti-human POT1 antibody (P0096, Sigma, 1:1,000 dilution), anti-GAPDH antibody conjugated to HRP (2118S; Cell Signaling Technology, Danvers, MA; 1:5,000 dilution), and secondary HRP-conjugated anti-rabbit IgG antibody (A0545, Sigma, 1:1,000 dilution) was used to detect the human POT1 antibody.

For the western blot analysis of YFP tagged WT and mutant POT1, standard immunoblot protocols were used with the following antibody dilutions: anti-GFP antibody (11814460001 Sigma, 1:2,000 dilution), anti-human POT1 antibody (ab21382 Abcam, 1:1,000 dilution), anti-GAPDH antibody conjugated to HRP (2118S Cell Signaling Technology; 1:5,000 dilution), and secondary HRP-conjugated anti-rabbit IgG antibody (A0545, Sigma, 1:1,000 dilution) was used to detect the human POT1 antibody.

Detection of antibody signal was done with chemiluminescence activated using 2 ml of Luminata Forte Western HRP Substrate (Millipore). The signal was detected and developed with a Fuji LAS-3000 scanner. Western blot signals were quantified using ImageQuant TL (GE Healthcare) and normalized using the β-Actin or GAPDH signal as a loading control.

### Reverse transcription PCR and quantitative PCR analyses

To test the effectiveness of the shRNA targeting the 3′UTR of endogenous POT1, we collected RNA from cells treated with shRNA by lysing pelleted cells in TRizol (Life Technologies) and purified RNA using the Direct-zol RNA mini prep kit (Zymo Research, Irvine CA). Reverse transcription PCR was performed using random hexamer primers followed by quantitative PCR using primers in 3′-UTR of POT1. The following primer pairs were used to measure human POT1 and GAPDH mRNA levels after knockdown: POT1 (5′CTCACCTTCCCTGTTTGAGCTT3′ and 5′TCCCATACCCATGCTAACATCA3′); GAPDH (5′ATGGAAATCCCATCACCATCTT3′ and 5′CGCCCCACTTGATTTTGG3′).

### Direct telomerase activity assays

Telomerase preparations were carried out as previous described by *Cristofari et al*^43^. Briefly, super telomerase extracts were prepared by lysing HEK293T cells overexpressing human TERT and TER (pcDNA6-hTERT and pBS-U1-hTER plasmids were a gift from J. Lingner) in CHAPS lysis buffer consisting of 150 mM KCl, 50 mM Hepes, pH 7.5, 1 mM MgCl_2_, 1 mM EDTA, 10% glycerol, 0.5% CHAPS, 5 mM beta-mercaptoethanol and 0.01 μl ml^−1^ of protease inhibitor cocktail (Sigma P8340). Direct assays were carried out with 150 nM of purified full-length POT1 or TPP1 incubated with 20 nM of A5 primer (TTAGGGTTAGCGTTAGGG) before the addition of 2 μg of super telomerase extract. In addition, direct assays were also performed with 5 μg of lysates from HEK293T+shPOT1 cells transiently transfected with 1 μg of pcDNA6-hTERT and 3 μg of pBS-U1-hTER plasmids. For transfections involving POT1 or TPP1, 1 μg of pLU-1xFlag-POT1 or pLU-1xHA-hTPP1 plasmid was used. In control transfections where POT1 or TPP1 were omitted, 1 μg of pLU-EF1A-iBlast (empty vector) was included.

Telomerase extension reactions were carried out in 20 μl at 30 °C for 60 min, 50 mM Tris–HCl, pH 8.0, 50 mM KCl, 1 mM spermidine, 1 mM MgCl_2_, 5 mm β-mercaptoethanol, 500 μM dATP, 500 μM dTTP, μM dGTP, 20 μCi of [α-^32^P]—dGTP (3,000 Ci mmol^−1^). Reactions were stopped with the addition of 100 μl of 3.6 M ammonia acetate, 20 μg of glycogen, 0.5 nM of 5′ ^32^P-labelled 18mer loading control, and were precipitated overnight with 500 μl of ethanol at −80 °C. The samples were pelleted by centrifugation at 14,000 RPM at 4 °C for 20 min, washed with 500 μl of 70% ethanol, centrifuged again for 10 min. The ethanol was removed from each sample and then air dried for 30 min. The pellets were then resuspended in a loading buffer containing 98% formamide, 1 mM EDTA, and 0.05% xylene cyanol, heated to 95 °C for 5 min, and then loaded onto a 10% acrylamide, 7 M urea, 1 × TBE sequencing gel. The gel was run for 3.5 h at 1,800 V, fixed with 30% Methanol 10% acetic acid solution and dried for 1 h with a gel dryer (Bio-Rad). The dried gel was then exposed to a phosphorous storage plate overnight and imaged the next day with a Typhoon 9410 Imager (GE Healthcare). Quantification of telomerase products was measured using ImageQuant TL software (GE Healthcare). Calculation of telomerase processivity was performed as described previously^9^,^44^. Briefly, each band was quantified using ImagequantTL (GE Healthcare) and the intensity was normalized against the loading control. Band intensity was then corrected for the number of radiolabeled nucleotides (hot Gs) added per repeat. The total lane counts (TLCs) were then measured by taking the sum of the normalized band intensity over the entire lane. Each corrected band was expressed as a fraction left behind (%LB) by summing the normalized intensity for each repeat and every repeat below it, divided by the total counts of every repeat in the lane (TLCs) and multiplied by 100. The natural log of (100-%LB) was then calculated and plotted for every repeat number. The plot of ln(100-%LB) per repeat number was fit with a linear regression equation with a slope *m*. The processivity was then calculated by taking –ln(2)/*m* for the slope of each line (Supplementary Fig. 2).

### Cell imaging

Human full-length TRF2 and WT and mutant POT1 genes were cloned into modified pEGFP-C3 vectors (BD Biosciences Clontech) carrying the YFP or mCherry N-terminal fusion tags. HEK 293 cells were transiently transfected with Cherry-TRF2 and YFP-POT1 plasmids using Fugene 6 (Promega, Madison WI) transfection for 24 h. Cells were fixed with freshly prepared paraformaldehyde (4% at room temperature) and stained with DAPI.

Confocal images were taken with a Leica SP8X white light laser scanning confocal microscope (Leica Microsystems, Buffalo Grove, IL) using a × 63 oil objective lens (numerical aperture 1.4) with × 6 zoom. For each cell, stacks of ∼15 images with 0.5 μm step sizes were acquired and processed using Huygens deconvolution software (Scientific Volume Imaging, The Netherlands). The files were then presented as maximum projection images using Leica LASX software.

### Southern blot

For telomere length analysis we carried out Southern blots on HEK293T cells stably expressing WT and mutant 1xFlag-POT1 and an shRNA targeting the 3′UTR of the endogenous POT1. Genomic DNA from cells at various passages was purified using a QIAamp DNA mini kit (Qiagen) and 10 μg of genomic DNA was then digested with AluI+MboI restriction endonucleases. 5′ ^32^P-labelled GeneRuler 1 kb Plus DNA ladder (Thermo) and 2 μg of digested DNA was then fractionated in a 0.7% agarose gel, denatured and transferred onto a GeneScreen Plys hybridization membrane (Perkin Elmer) overnight. The membrane was cross-linked, hybridized at 42 °C with 5′-end-labaled ^32^P-(TTAGGG)_4_ probe in Church buffer (0.5 N Na2HPO4, pH 7.2, 7% SDS, 1% BSA, 1 mM EDTA) overnight and then washed three times for 20 min each with wash buffer (0.2 M Na2HPO4, pH 7.2, 1 mM EDA, and 2% SDS) at 37 °C. The membrane was exposed to a phosphorous storage plate overnight and visualized by Typhoon 9410 Imager (GE Healthcare). Telomere length was calculated using the software TeloTool (MATLAB)^45^.

### Fluorescence *in situ* hybridization

HEK293T cells stably expressing WT and mutant 1xFlag-POT1 and an shRNA targeting the 3'UTR of the endogenous POT1 were passaged 10 times (∼50 population doublings) and grown to 40% confluence on a 10 cm plate and treated with 100 μg ml^−1^ of colcemid for 4 h. Cells were then trypsinized to detach them from the plate, pelleted and treated in a hypertonic environment (75 mM KCl for 30 min at 37 °C). Cells were then fixed in 10 ml of 3:1 methanol:acetic acid solution and stored at 4 °C. Cells were dropped on frosted microscope slides (Thermo Scientific), washed with 3:1 methanol:acetic acid solution, heated for 5 min at 70 °C, and dried overnight. Slides were then rehydrated in coplin jars with PBS, fixed with 4% formaldehyde (Sigma), washed 3 times with PBS and dehydrated in 70, 95, and 100% ethanol successively. Slides were then air dried and hybridized with 20 ul of 200 nM TelC-Tamra peptide nucleic acid (PNA) probe ((CCCTAA)_3_-Tamra, Panagene) in 70% formamide, 10 mM Tris pH 7.5, 0.5% Odyessy blocking buffer (LiCor) according to the manufacturerαs instructions. Slides were stained with DAPI and imaged using a Nikon 80i upright microscope using a × 100 oil objective.

### Data availability

The atomic coordinates and structure factors for the POT1C-TTP1 complex described here have been deposited in the Protein Data Bank under the accession code 5UN7. The data that support the findings of this study are available from the corresponding author on request.

## Additional information

**How to cite this article:** Rice, C. *et al*. Structural and functional analysis of the human POT1-TPP1 telomeric complex. *Nat. Commun.* **8**, 14928 doi: 10.1038/ncomms14928 (2017).

**Publisher's note:** Springer Nature remains neutral with regard to jurisdictional claims in published maps and institutional affiliations.

## Supplementary Material

Supplementary Information Supplementary Figures and Supplementary Tables

## Figures and Tables

**Table 1:** Data collection, phasing and refinement statistics.

	**Native (Zn data)**	**Phasing Hg derivative 1**
*Data collection*		
Wavelength (Å)	1.0332	1.0000
Space group	P4_1_22	P4_1_22
Cell dimensions		
*a*, *b*, *c* (Å)	99.5 99.5 122.1	99.0 99.0 124.2
Resolution (Å)	38.6−2.1 (2.15−2.1)*	40–3.00 (3.08–3.00)
*I*/s*I*	22.8 (2.7)	12.3 (3.5)
Completeness (%)	99.8 (100)	99.9 (100)
Redundancy	31.3 (31.4)	7.6 (7.6)
		
*Phasing analysis*		
Resolution (Å)		40−3.00
Number of sites		5
Pseudo-free CC:		68.2
Mean figure of merit (FOM)		0.605
		
*Refinement*		
Resolution (Å)	20-2.1 (2.16−2.10)	
No. reflections	34,593 (2,473)	
*R*_work/_ *R*_free_	18/22 (20.8/25.5)	
No. atoms		
Protein	2,858	
Water	531	
B-factors		
Protein	40.4	
Water	54.6	
R.m.s deviations		
Bond lengths (Å)	0.008	
Bond angles (°)	1.233	
Ramachandran plot (%)		
Most favoured	98.9	
Allowed	1.1	

^*^Highest resolution shell is shown in parenthesis.
