# Supplementary material for: Structural and functional analysis of the human POT1-TPP1 telomeric complex
Source: Nat Commun. 2017 Apr 10;8:14928. doi: 10.1038/ncomms14928 (PMC5394233; doi:10.1038/ncomms14928)
Supplement: Supplementary Information — Supplementary Figures and Supplementary Tables [file ncomms14928-s1.pdf]

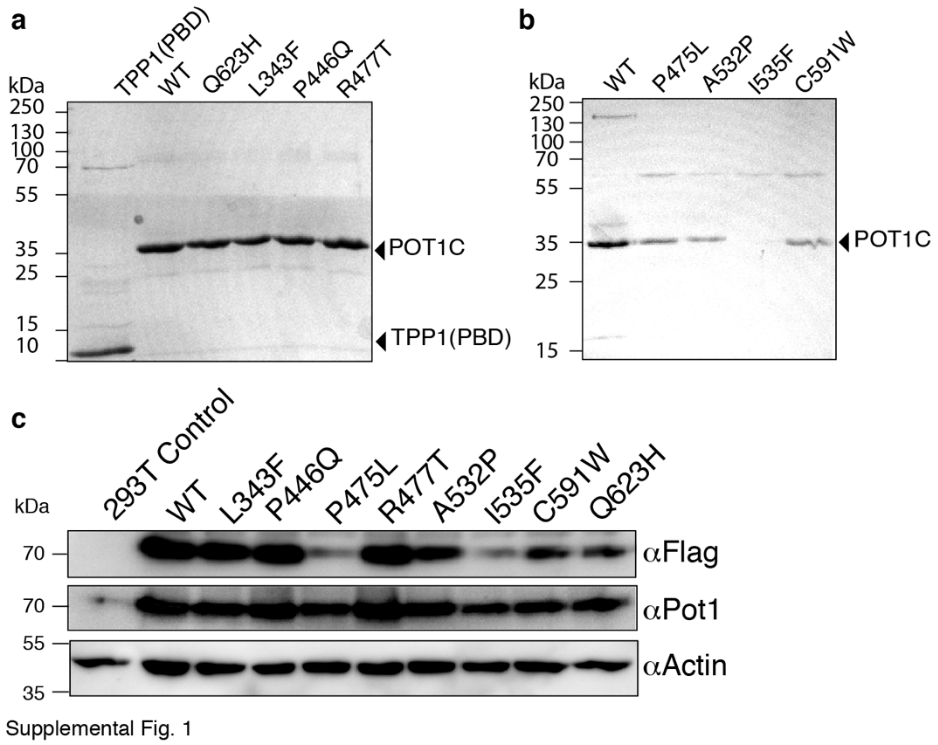

Supplementary Fig. 1. (**a**) SDS-PAGE of purified TPP1(PBD) and WT and Q623H, L343F, P446Q and R477T mutant POT1C proteins used in ITC experiments. (**b**) SDS-PAGE of WT and P475L, A532P, I535F, and C591W mutant POT1C used in ITC experiments. Arrows indicate POT1C and TPP1(PBD). The POT1C mutant I535F protein levels are significantly lower compared to the WT protein suggesting that the protein is not stable when over-expressed at least in the absence of TPP1. (**c**) Western blot showing expression levels of full-length WT and mutant Flag-POT1 proteins after transient transfection of HEK293T cells.

a

Band#	Volume	Repeat#	HotsGs	Corrected Vol.	%LB	ln(100-%LB)
1	8099.63	20	60	134.99	100.00	#NUM!
2	11818.39	19	57	207.34	99.59	-0.8926915
3	10057.74	18	54	186.25	98.96	0.03786633
4	10412.86	17	51	204.17	98.40	0.472289786
5	9178.25	16	48	191.21	97.78	0.798900812
6	10933.92	15	45	242.98	97.20	1.030765434
7	9038.86	14	42	215.21	96.46	1.264229845
8	7712.98	13	39	197.77	95.81	1.433484293
9	8610.73	12	36	239.19	95.21	1.567216348
10	9013.97	11	33	273.15	94.48	1.708186868
11	11884.77	10	30	396.16	93.65	1.848083987
12	11949.48	9	27	442.57	92.45	2.021485987
13	14845.17	8	24	618.55	91.11	2.185178961
14	18968.83	7	21	903.28	89.23	2.376655711
15	22091.86	6	18	1227.33	86.49	2.603374175
16	27504.88	5	15	1833.66	82.77	2.846813033
17	29190.85	4	12	2432.57	77.20	3.126577789
18	36293.17	3	9	4032.57	69.82	3.407043858
19	40919.63	2	6	6819.94	57.59	3.747388063
20	36487.32	1	3	12162.44	36.90	4.14473483

b

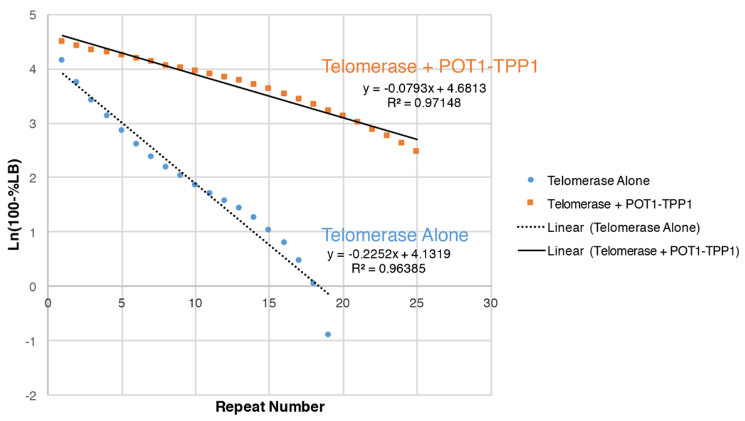

Supplementary Fig. 2. Calculation of telomerase processivity. Each band in Figure 4C and d was quantified using ImagequantTL (GE Healthcare) and the intensity was normalized against the loading control. Band intensity was then corrected for the number of radiolabeled nucleotides (hot Gs) added per repeat. The total lane counts (TLCs) were then measured by taking the sum of the normalized band intensity over the entire lane. Each corrected band was expressed as a fraction left behind (%LB) by summing the normalized intensity for each repeat and every repeat below it, divided by the total counts of every repeat in the lane (TLCs) and multiplied by 100. The natural log of (100-%LB) was then calculated and plotted for every repeat number. The plot of ln(100-%LB) per repeat number was fit with a linear regression equation with a slope m. The processivity was then calculated by taking $-\ln(2)/m$ for the slope of each line (Supplemental Fig.

.

Reported Cases	POT1C Mutation	Reported Cancer Type	Mutation Type	References
1 case	L343F	Chronic Lymphocytic Leukemia (CLL)	Somatic, Heterozygous	Ramsay, A. J. et al.
1 case	S421L	Melanoma	Germline	Shi, J. et al.
1 case	L454S	Melanoma	Germline	Shi, J. et al.
1 case	I455V	Melanoma	Germline	Shi, J. et al.
1 case	P446Q	CLL	Somatic	Ramsay, A. J. et al.
1 family, 3 cases	E450X	Glioma	Germline; nonsense mediated decay	Bainbridge et al.
1 case	K465*	CLL	Somatic, nonsense mutation	Ramsay, A. J. et al.
1 case	P475L	CLL	Somatic, Heterozygous	Ramsay, A. J. et al.
1 case	R477T	CLL, Non-small cell lung cancer	Somatic	Ramsay, A. J. et al.
2 families, 4 cases	A532P	Melanoma	Germline	Shi, J. et al.
1 case	I535F	CLL, Lung adenocarinoma	Somatic	Ramsay, A. J. etal.
1 case	C591W	CLL	Somatic, Heterozygous	Ramsay, A. J. et al.
1 family, 6 cases	Splice acceptor-variant (between exon 17/18)	Melanoma	Germline	Robles-Espinoza et al.
1 family, 1 case	D617Efs	Glioma	Germline; frameshift	Bainbridge et al.
1 family, 2 cases	Q623H	Melanoma	Germline	Shi, J. et al.

Supplementary Table 1. Table of reported POT1C associated cancer mutations in the cBIOPortal for cancer genomics (http://cbioportal.org/), the COSMIC catalog of somatic mutations in cancer (http://cancer.sanger.ac.uk/), and published literature.

Protein	TPP1-PBD	POT1C							
	WT	WT	L343F	P446Q	P475L	R477T	A532P	C591W	Q623H
kD (nM)		120	114	289	164	123	117	870	472
SD (nM)		±16	±13	±15	±32	±20	±13	±226	±142

Supplementary Table 2. Table of ITC binding data for WT and mutant POT1C and TPP1(PBD).

Protein	POT1C	TPP1(PBD)	POT1C-TPP1(PBD)	TPP1(87)	Full-length POT1			
	WT				WT	P446Q	C591W	Q623H
kD (nM)	Undetermined				19.8	18.0	18.6	19.7
SD (nM)					±4.0	±4.6	±3.6	±4.1

Protein	Full-length POT1-TPP1(87) Complex							
	WT	L343F	P446Q	P475L	R477T	A532P	C591W	Q623H
kD (nM)	5.8	4.9	10.3	10.7	5.2	5.3	15.6	8.9
SD (nM)	±0.5	±0.5	±1.0	±1.4	±0.5	±0.6	±2.5	±1.0

Supplementary Table 3. Table of FP assay data for WT and mutant full-length POT1 and WT TPP1(87) proteins with the telomeric 18mer. SD denotes standard deviation.
